# “Contemporary Treatment in Multi-Professional Team of Acute Psychosis- Pros and Cons”

**DOI:** 10.1192/j.eurpsy.2022.189

**Published:** 2022-09-01

**Authors:** J. Jaudzema

**Affiliations:** Riga Centre of Psychiatry and addiction disoders, Psychiatry, Rīga, Latvia

**Keywords:** psychiatry, early intervention, acute psychosis, Multi-Professional Team

## Abstract

The approach of a multi-professional team in the treatment of acute psychosis was started in 2018/2019 In Latvia, within the framework of the early intervention program. Until then, the patient was mostly examined and treated with medication in the inpatient phase of acute psychosis. After discharge, further treatment and rehabilitation in Latvia is still relatively limited and largely based on the initiative of the patient and relatives. Within the multi-professional team of the inpatient department of the Riga Psychiatry and Narcology Center, a psychiatrist, mental health nurses, nursing assistants, psychologists, occupational therapists, a physiotherapist, a visual arts and dance - movement therapist, as well as a social worker work together. The benefits of teamwork in the treatment of acute psychosis are undeniable, but during these few years, working in challenging conditions, various disadvantages have crystallized. Among the ’pros’ is a more successful, multifaceted outcome of recovery, better patient compliance and reduction of stigma among patients and their relatives (and thus in society as a whole). ’Cons’ are mainly due to limited availability of resources, from limited funding and infrastructure to a lack of professionals and time. It should also be noted that mental health literacy is still low in Latvian society, which means that patients often arrive late for help, which means that it is not possible to achieve good results in a limited time within the hospital. For these reasons, treatment is often discontinued after discharge, which increases the risk of rehospitalisation and adverse course of the disease.

**Disclosure:**

No significant relationships.

